# Electromagnetically induced transparency metamaterial based on spoof localized surface plasmons at terahertz frequencies

**DOI:** 10.1038/srep27596

**Published:** 2016-06-09

**Authors:** Zhen Liao, Shuo Liu, Hui Feng Ma, Chun Li, Biaobing Jin, Tie Jun Cui

**Affiliations:** 1 State Key Laboratory of Millimeter Waves, Department of Radio Engineering, Southeast University, Nanjing 210096, China; 2 Synergetic Innovation Center of Wireless Communication Technology, Southeast University, Nanjing, 210096, China; 3 Cooperative Innovation Centre of Terahertz Science, No. 4, Section 2, North Jianshe Road, Chengdu 610054, China; 4 Research Institute of Superconductor Electronics, School of Electronics Science and Engineering, Nanjing University, Nanjing 210093, China

## Abstract

We numerically and experimentally demonstrate a plasmonic metamaterial whose unit cell is composed of an ultrathin metallic disk and four ultrathin metallic spiral arms at terahertz frequencies, which supports both spoof electric and magnetic localized surface plasmon (LSP) resonances. We show that the resonant wavelength is much larger than the size of the unit particle, and further find that the resonant wavelength is very sensitive to the particle’s geometrical dimensions and arrangements. It is clearly illustrated that the magnetic LSP resonance exhibits strong dependence to the incidence angle of terahertz wave, which enables the design of metamaterials to achieve an electromagnetically induced transparency effect in the terahertz frequencies. This work opens up the possibility to apply for the surface plasmons in functional devices in the terahertz band.

In the past decades, surface plasmons (SPs) have been extensively investigated in the optical frequencies^1^. Due to their extremely strong and confined optical fields, SPs have shown various potential applications in the photonic circuits^2^,^3^, near-field microscopy^4^,^5^, biological sensors^6^,^7^,^8^, photovoltaics^9^,^10^, etc. Since the spoof (or designer) surface plasmon polaritons (SPPs) allow a route to imitate the optical SPPs^11^,^12^, a variety of advanced plasmonic researches have been put forward and experimentally realized at low frequencies ranging from the microwave to infrared spectra, such as conformal surface plasmons waveguide^13^, negative-index waveguide^14^, and terahertz SPPs^15^,^16^,^17^,^18^. Recently, an important discovery showed that periodically textured perfectly electrical conducting (PEC) particles either in two or three-dimensions is able to mimic the natural localized surface plasmons (LSPs) at lower frequencies, which is called as spoof LSPs^19^. More recently, an ultrathin plasmonic spoof LSP resonator has been proposed, which is easy for fabrication and integration^20^. Based on the design of these plasmonic metamaterial particles, the conventional LSPs at optical frequencies can be directly scaled down to lower frequencies^21^.

Apart from the features of conventional LSPs shown in the optical frequencies, many phenomena have been predicted and experimentally verified for spoof LSPs, such as the vertical transportation^22^, unusual electric and magnetic LSPs resonances^23^ and high-order electric LSPs resonances^24^,^25^. However, all earlier investigations and experiments on spoof LSPs are limited to the microwave frequency. With the rapid development of terahertz technology in recent years, the implementation of terahertz spoof LSPs resonators may become possible, which will facilitate the device miniaturization and system integration in the terahertz band.

In this article, we propose the LSP particle in the terahertz frequency using ultrathin spirally corrugated metallic disk and generate the LSP resonances. We demonstrate that both spoof electric and magnetic LSPs are supported by the proposed ultrathin structure in the terahertz band, which is in deep-subwavelength scale and highly relies on the incident angle of terahertz waves. By slightly tuning the geometrical parameters of the proposed structure, we could change the resonant frequencies and further increase the subwavelength level. Besides the geometrical parameters, arrangements and separations have also significant effects on the resonance frequency^26^,^27^. These approaches extends the capabilities of spoof LSPs, and can be applied to the terahertz integrated circuits. Using the proposed magnetic LSPs, we further construct an electromagnetically induced transparency (EIT) metamaterial in the terahertz spectrum^28^,^29^. Numerical simulations, experimental measurements are conducted to evaluate and validate the performance of the proposed spoof LSPs, promising in a number of potential terahertz applications such slow lights, filters, and sensing.

## Results

**Figure 1:**
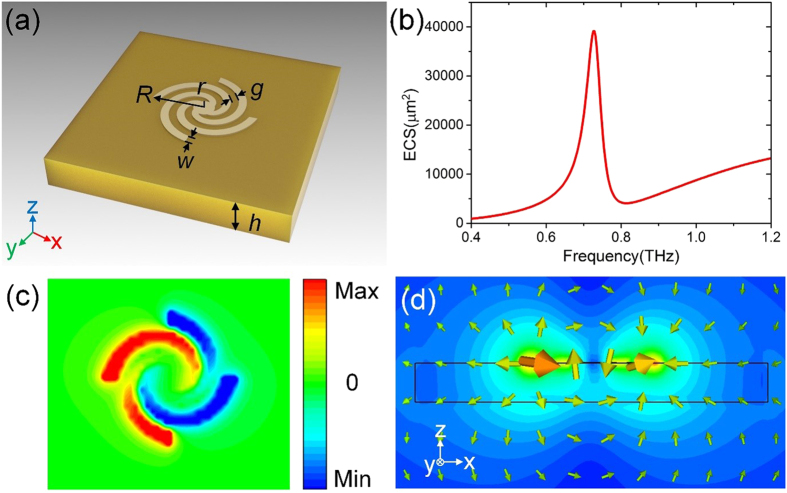
(**a**) Schematic diagram of the ultrathin metallic spiral structure. (**b**) The calculated ECS spectra at normal incidence. (**c**) The simulated *E*_*z*_ near-field pattern at the resonance frequency. (**d**) The magnitude of the electric fields at the resonance frequency, in which the arrows show the electric field lines.

The structure of the designed LSPs particle is schematically illustrated in Fig. 1(a), which is composed of four gold spiral arms and one internal gold disk with ultrathin thickness 0.2 μm. The radius *r* of the inner disk and length *R* of the outer spiral arms are 6.5 μm and 33 μm, respectively. The four spiral arms have the same linewidth *w* = 5 μm, and are separated by a gap *g* = 5 μm. The designed LSPs particle is fabricated on a polyimide substrate with 20 μm thickness (*ε* = 3, *tan* = 0.03).

We first consider the case when the particle is normally illuminated with an *x*-polarized plane wave. The extinction cross sections (ECSs) are calculated using commercial software, the CST Microwave Studio 2013, as shown in Fig. 1b. Here, the ECSs are sums of absorption cross sections and scattering cross sections. In Fig. 1b, a peak can be clearly observed at 0.73 THz. To gain more insights into the resonance, we give the simulated electric field distributions (*E*_*z*_) in the *x-y* plane 5 μm above the metallic structure at 0.73 THz, as shown in Fig. 1c. The electric fields distribution matches well with the previously reported spoof LSPs in the microwave frequency^23^. Meanwhile, we plot in Fig. 1d the magnitude of the electric fields and electric field lines of this mode in a cross section *y* = 0. We can see that the arrows go from left of the structure to right. Clearly, this mode profiles correspond to the electric-dipole mode, indicating that the proposed structure supports spoof LSPs resonance at terahertz frequency.

**Figure 2:**
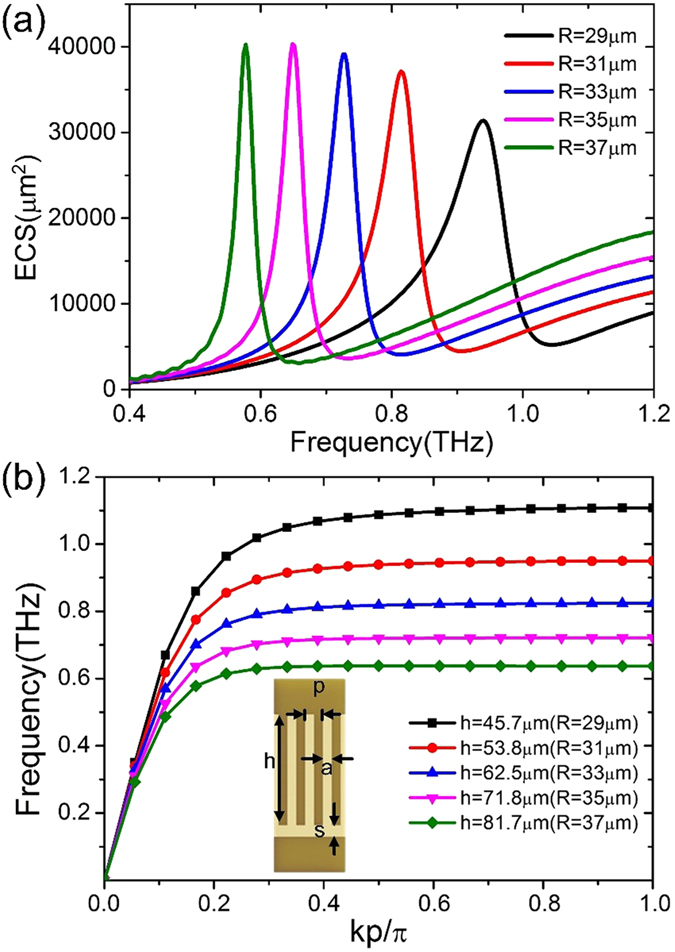
(**a**) The simulated ECS spectra with respect to different outer radius. (**b**) The dispersion relations of spoof SPPs with different depth. The inset shows the schematic picture of spoof SPPs strip.

In Fig. 2(a), we show the calculated ECS spectra as a function of the outer radius *R*. When the radius *R* of the particle increases from 29 to 37 μm, the resonance frequency experiences an obvious redshift from 0.94 to 0.577 THz. To reveal the reason for the redshift in the proposed spoof LSP resonator, we first investigate the nature of spoof SPPs, since the spoof LSP resonator arises from the spoof SPP strip and the work frequency of the spoof SPPs has many influences on the resonance frequency of the relevant spoof LSPs. Textured surfaces can support spoof SPs due to the cavity-waveguide mode inside the groove, which is analogous to the penetration of fields into the metal. Either in straight or spiral groove, the frequency of existing SPs depends mainly on the depth of grooves. Here, although grooves in the proposed structure are spiral, it has been proved that the spiral grooves behave like the straight grooves approximatively^23^. The mentioned spoof SPP structure is illustrated in the inset of Fig. 2(b), whose parameters are set as *s* = 6.5 μm, *p* = 10 μm, and *a* = 5 μm. The depths of the straight grooves *h* are equal to the lengths of spiral curves in spoof LSP resonators, which are 45.7 μm, 53.8 μm, 62.5 μm, 71.8 μm, and 81.7 μm according to *R* = 29 μm, 31 μm, 33 μm, 35 μm, and 37 μm. The thicknesses of the metal and substrate are 0.2 μm and 20 μm, respectively. The calculated dispersion relations of spoof SPPs are presented in Fig. 2(b), from which we obtain that the deeper grooves correspond to the lower asymptotic frequencies. Actually the spoof LSP is a standing wave on the spoof LSP loop. Meanwhile because deeper grooves have lower frequencies for the same wavenumber, as shown in Fig. 2(b), hence the spoof LSP resonators composed of deeper grooves have lower resonance frequencies. Here, the deeper groove means longer spiral curve in the particle, which represents that the particle has bigger radius. Therefore, the resonance frequencies get smaller as the radiuses become bigger, as plotted in Fig. 2(a).

**Figure 3:**
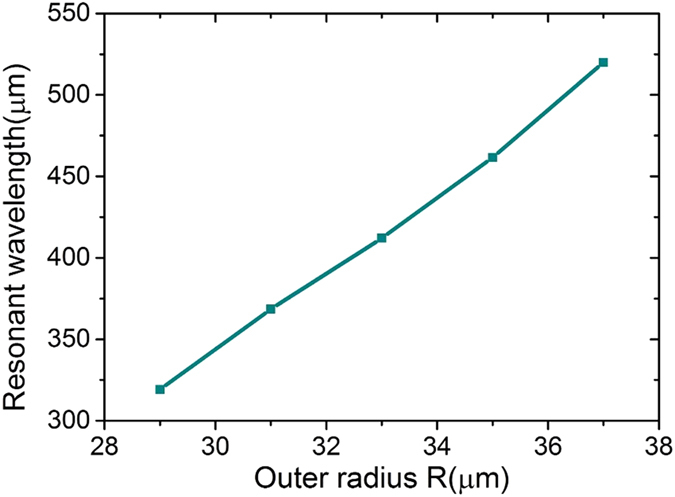
The resonant wavelengths of metamaterial particle as a function of the radius *R*.

In addition, we note that the radius *R* is much less than the resonant wavelength in the present design, making it a deep subwavelength structure. To gain an intuitional insight into their relationship, Fig. 3 further gives the resonance wavelengths *λ*_*res*_ as a function of the outer radius *R*, in which we see that the resonance wavelengths increase from about 319 to 520 μm as *R* increases from 29 to 37 μm. It can be calculated that a 27% change in size (from *R* = 29 μm to *R* = 37 μm) results in a 201 μm (or 63%) increase in resonance wavelength, demonstrating that *λ*_*res*_ can be tuned in large range by simply varying the radius *R*. Most importantly, by calculating the equivalent electrical length of the case when *R* equals 29 and 37 μm, we found that the bigger the radius R, the more the deep subwavelength effect is. This can be attributed to the large electrical length formed by the spiral grooves of particle, which effectively reduce the resonant frequency. Moreover, small change in radius *R* will result in great variation in the effective length of spiral grooves.

**Figure 4:**
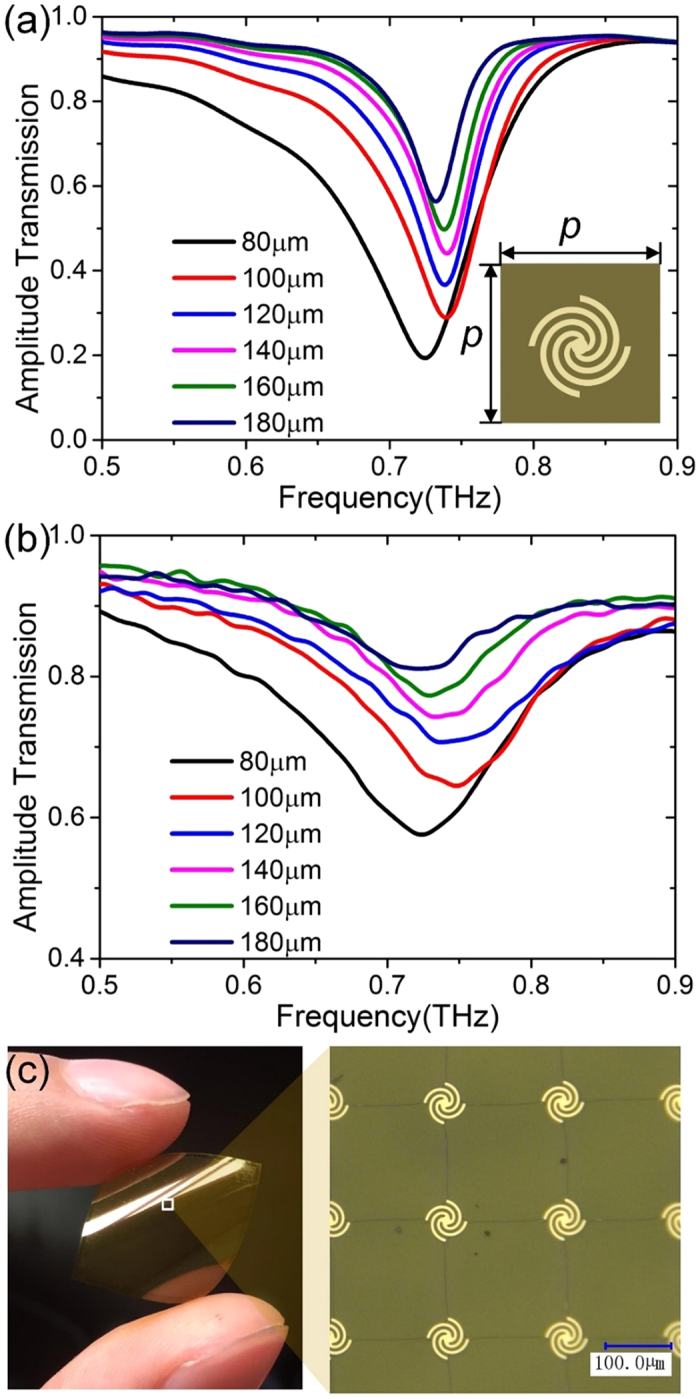
(**a**) The simulated amplitude transmission spectra for the two-dimensional array with lattice constants *p* from 80 to 180 μm. (**b**) The measured amplitude transmission spectra for the two-dimensional array with lattice constants *p* from 80 to 180 μm. (**c**) The photo of sample and microscopic image of the fabricated structure.

The resonance frequency is not only highly dependent on the size of the meta-atom, but also depends on arrangement due to the electromagnetic interactions between neighbor particles. To evaluate these interactions, we simulate the transmission spectra using periodic boundaries. Figure 4(a) shows the results for square arrays of meta-atoms with lattice constant *p* = 80–180 μm. As array spacing decreases, the resonance frequency first blue shifts for *p* larger than 80 μm and then redshifts for smaller distances.

To analyze the unit coupling effects, we introduce a simple semi-analytical model which can help understand this trend qualitatively^26^,^27^. In this model, the retarded dipole sum *S* can reveal the variation of the spectra with arranged period. It was demonstrated that the real parts of *S* imply the resonant frequency shifts. The positive value corresponds to red shift of *λ*_*max*_, while the negative value represents blue shift. This sum *S* is given by


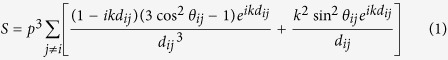


where *d*_*ij*_ is the distance between the *i*th and *j*th particles, *θ*_*ij*_ is the angle between the vector from units *i* to *j* and the polarization vector. From Fig. 4(a), we find that the resonant frequencies are approximately 0.75 THz (400 μm). Then we calculate the values of *S* as the period *p* changes from 180 to 100 μm for *λ *= 400 μm. The results are presented in Table 1.

Table 1 demonstrates that the real part of *S* is negative when *p* changes from 180 to 100 μm, while is positive for *p* = 80 μm. The real part becomes less negative as the period *p* decreases and becomes positive until *p* = 80 μm. In other words, blue shifts occur for *p* = 180 to 100 μm and red shifts for *p* = 80 μm, which have good agreement with the trend in Fig. 4(a). Actually, the expression for *S* can be divided into two parts, one of which has 1/*d* component, representing the radiative dipole interaction, and the other of which has 1/*d*^*3*^ component, showing the static dipole coupling. Hence we find that for big lattice constant the radiative dipole interaction is dominant, whereas the static dipole coupling plays a more important role as the period *p* decreases. And the resonance blue shifts (negative value in real parts of *S*) are the result of the radiative dipole interaction for bigger separations, whereas red shifts due to the static dipole coupling for smaller lattice spacing.

To experimentally verify the performance of the proposed spoof LSPs arrays, we fabricate samples, whose unit cells are distributed on a two-dimensional surface with lattice constants *p* from 80 to 180 μm. Each sample occupies an area of 15 × 15 mm, which is wide enough to cover the focused THz beam in experiments. Figure 4(c) illustrate the sample with *p* = 180 μm and his zoomed image taken by an optical microscope (VHX-5000, Keyence Company). The measured transmission spectra are plotted in Fig. 4(b). In the measured spectra, the resonance wavelength first blue shifts and then red shifts, the switch in behavior occurring at 80 μm, which match with the trend in Fig. 4(a). Both calculation and experiment demonstrate that meta-atom arrangement and separation play an important role in resonance frequency.

**Figure 5:**
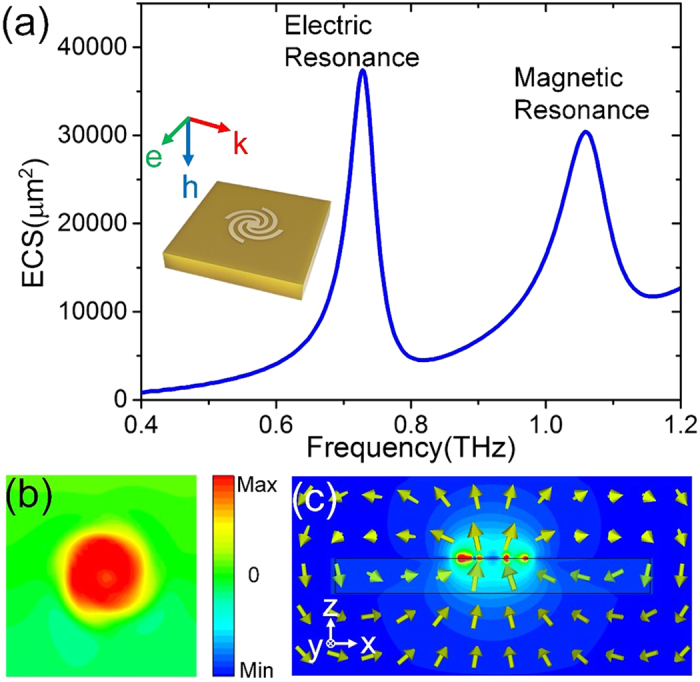
(**a**) The calculated ECSs spectra at grazing incidence. (**b**) The simulation result of *H*_*z*_ near-magnetic-field distribution corresponding to the magnetic resonance. (**c**) The magnitude of the magnetic fields at the resonance frequency. The arrows show the magnetic field lines.

So far, magnetic resonance is not observed in the proposed structure under the normal incidence. Then we proceed to calculate the extinction cross sections (ECSs) of a single meta-atom under grazing incidence, aiming to excite magnetic resonance, shown in Fig. 5(a). The ECSs spectrum shows two distinct peaks, and this phenomenon closely resembles that discovered at microwave frequencies in previous published works^23^,^25^. The lower frequency at 0.73 THz corresponds to the electric resonance, which is same to the frequency of the resonance peak under the normal incidence. Then we plot the magnetic field distribution (*H*_*z*_) of the higher peak at 1.06 THz in Fig. 5(b). The magnetic field distribution is similar to a magnetic dipole. To confirm this mode, the magnitude of the magnetic fields and magnetic field lines in transverse plane *y* = 0 are simulated, shown in Fig. 5(c). The *H*-field lines is circulating around the disk, and thus the mode was termed as magnetic LSPs.

**Figure 6:**
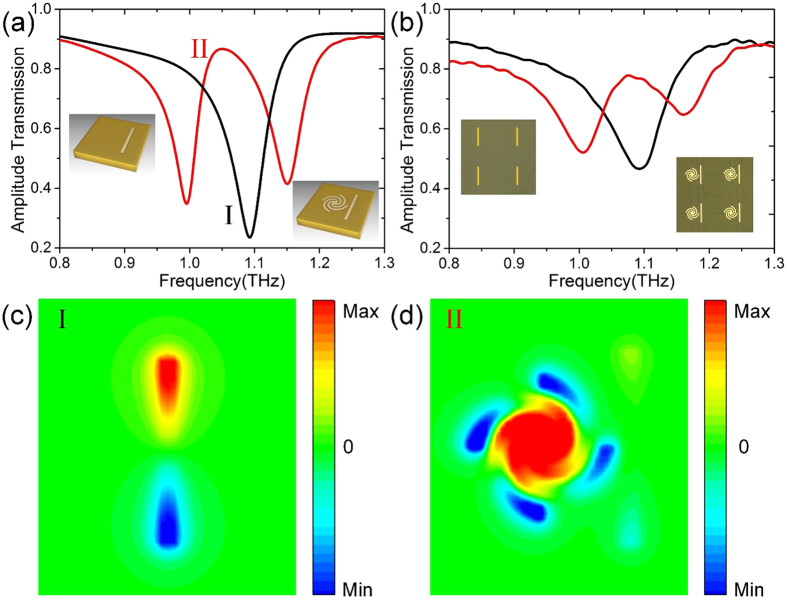
(**a**) The simulated amplitude transmission spectra of the sole-wire (the black line), and the EIT metamaterial (the red line). The insets present the structure of the simulated unit cell. (**b**) The measured amplitude transmission spectra of the sole-wire (the black line), and the EIT metamaterial sample (the red line). The insets present the microscopy images of the fabricated sample. The simulated electric field (*E*_*z*_) distributions of (**c**) the sole-wire and (**d**) the EIT metamaterial at 1.07 THz.

We have demonstrated that for spoof LSPs resonance, normal incidence only excite the electric LSPs mode directly while the magnetic LSPs mode needs to be generated by grazing incidence. By this feature, magnetic LSPs mode is able to serve as EIT dark element. Generally, EIT spectral response is attributed to the destructive interference between bright and dark mode resonances, which corresponds to broad resonance and sharp resonance, respectively. Therefore, a metallic wire with length 90 μm and linewidth 5 μm is used to generate bright mode, as displayed in the left inset of Fig. 6(a). These wires are distributed on a polyimide substrate with lattice constant *p* = 180 μm. The simulated transmittance of the wires array under y-polarized (along the wire) normally incident terahertz wave are given in Fig. 6(a) (black curve). The wires array show a low quality factor and broad resonance. To visualize this resonance mode, Fig. 6(c) plots the simulated *E*_*z*_ near-field distribution at 1.07 THz, which shows a typical dipolar resonance. In this case, the metallic wires are strongly coupled to the normal incident wave, which enable them to be bright mode.

Next, we place the straight wires close to the spoof LSPs resonators with distance of 5 μm, as shown in the right inset of Fig. 6(a). In this case, the normal terahertz waves can drive the wires directly while the spoof magnetic LSPs resonators have no response. However, the near field coupling between them excites the magnetic resonance in the spoof LSPs resonator. The destructive interference between the dipole and magnetic LSPs resonances results in a transparency window at 1.07 THz in the simulated transmission spectra, as illustrated in Fig. 6(a) (the red line). To visually verify the strong EIT effect arising from destructive interference, the simulated *E*_*z*_ near-field distribution of the unit cell is shown in Fig. 6(d), in which strong spoof magnetic LSPs resonance can be clearly observed^23^,^25^. Comparing with Fig. 6(c), the dipole resonance in Fig. 6(d) is very weak due the destructive interference effect. In order to experimentally verify the EIT behavior, a sample combining both wires and LSPs resonators is fabricated, as shown in the inset of Fig. 6(b). The experimental results of transmissions presented in Fig. 6(b), which are in good agreement with the simulation results. However, one may notice that the quality factor of the measured spectra is lower than simulation, which can be also observed in Fig. 4(b). This discrepancy is most likely due to surface roughness and the higher loss of metallic structure in the fabricated sample.

## Conclusion

In summary, we have numerically and experimentally demonstrated that spoof electric and magnetic LSPs can be realized at terahertz by metamaterial. The dimension of the meta-atoms is much smaller than resonance wavelength, making the proposed structure to be deep subwavelength scale. Both dimensions and arrangements can affect the resonance frequency. It is found that a sharp transparent transmission window can be achieved by placing the spoof LSPs resonator close to a metallic cut wire, between which destructive interference occurs. This work not only offers a way to obtain spoof LSPs resonance at terahertz frequency, but also make use of it to exhibit a EIT effect. Such plasmonic metamaterials may find interesting applications in plasmonic devices at terahertz frequency.

## Methods

### Manufacturing method

The detailed fabrication processes are described as follows. First, a 20 μm thick polyimide is coated to a silicon wafer as the substrate. Then, it was cured on hotplate at 80, 120, 180 and 250 °C for 5, 5, 5 and 20 minutes, respectively. A standard photolithography was then conducted before depositing a Ti/Au layer (10/180 nm) with electron beam evaporation, after which a lift-off process helped form the final metallic pattern. Finally, we peeled off the sample from the silicon wafer by immersing it in HF solvent for 30 minutes.

### Simulation method

All numerical simulations are performed by the commercial software, CST Microwave Studio 2013. The relevant substrate is polyimide, whose permittivity and loss tangent are 3 and 0.03, respectively. The metal is chosen as gold, which is selected in the material library in CST. For ECS calculation, a plane-wave excitation and a broadband far-field monitor are applied to record absorption cross sections and scattering cross sections. The transmissions are simulated by the CST transient solver, in which the periodic boundary conditions are used. The calculation of dispersion relation is based on the CST eigenmode solver, in which the eigen frequencies of a unite cell is analyzed.

### Experimental setup and measurement

**Figure 7:**
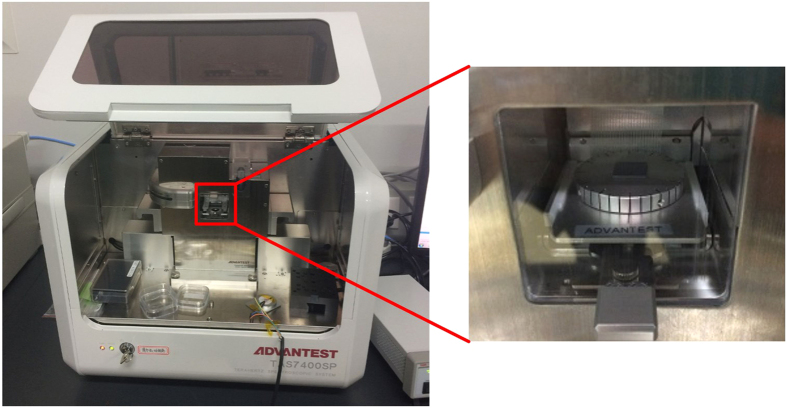
Photograph of the experimental setup used to measure the transmission of samples.

The experimental setup is a commercial Terahertz Spectroscopic Systems (TAS7400SP). The samples are placed on a platform, as shown in Fig. 7. All the experiments were measured in nitrogen environment (humidity smaller than 1%) under 25 °C. All spectra are normalized to the transmission without sample.

## Additional Information

**How to cite this article**: Liao, Z. *et al*. Electromagnetically induced transparency metamaterial based on spoof localized surface plasmons at terahertz frequencies. *Sci. Rep*. **6**, 27596; doi: 10.1038/srep27596 (2016).

## Figures and Tables

**Table 1:** The dipole sum *S* and its two components for λ = 400 μm.

p(μm)	S	S(1/d^3^)	S(1/d)
80	0.52 + 6.04i	3 + 3.61i	−2.48 + 2.43i
100	−2.71 + 7.8i	2.21 + 4.29i	−4.92 + 3.54i
120	−4.49 + 6.86i	1.23 + 4.8i	−5.72 + 2.06i
140	−8.27 + 7.19i	0.15 + 5.14i	−8.43 + 2.05i
160	−10.58 + 4.85i	−1.04 + 5.25i	−9.54-0.4i
180	−14.06 + 2.91i	−2.3 + 5.1i	−11.76-2.2i

## References

[b1] Maier S. A. Plasmonics: Fundamentals and Applications: Fundamentals and Applications. (Springer Science & Business Media, 2007).

[b2] Barnes W. L., Dereux A. & Ebbesen T. W. Surface plasmon subwavelength optics. Nature 424, 824–830 (2003). 12917696 10.1038/nature01937

[b3] Ozbay E. Plasmonics: Merging Photonics and Electronics at Nanoscale Dimensions. Science 311, 189–193 (2006). 16410515 10.1126/science.1114849

[b4] Kawata S., Inouye Y. & Verma P. Plasmonics for near-field nano-imaging and superlensing. Nat Photon 3, 388–394 (2009).

[b5] Gramotnev D. K. & Bozhevolnyi S. I. Plasmonics beyond the diffraction limit. Nat Photon 4, 83–91 (2010).

[b6] Anker J. N. . Biosensing with plasmonic nanosensors. Nat Mater 7, 442–453 (2008). 18497851 10.1038/nmat2162

[b7] Liu N., Mesch M., Weiss T., Hentschel M. & Giessen H. Infrared Perfect Absorber and Its Application As Plasmonic Sensor. Nano Lett. 10, 2342–2348 (2010). 20560590 10.1021/nl9041033

[b8] Larsson E. M., Alegret J., Käll M. & Sutherland D. S. Sensing Characteristics of NIR Localized Surface Plasmon Resonances in Gold Nanorings for Application as Ultrasensitive Biosensors. Nano Lett. 7, 1256–1263 (2007). 17430004 10.1021/nl0701612

[b9] Atwater H. A. & Polman A. Plasmonics for improved photovoltaic devices. Nat Mater 9, 205–213 (2010). 20168344 10.1038/nmat2629

[b10] Schuller J. A. . Plasmonics for extreme light concentration and manipulation. Nat Mater 9, 193–204 (2010). 20168343 10.1038/nmat2630

[b11] Pendry J. B., Martín-Moreno L. & Garcia-Vidal F. J. Mimicking Surface Plasmons with Structured Surfaces. Science 305, 847–848 (2004). 15247438 10.1126/science.1098999

[b12] Garcia-Vidal F. J., Martín-Moreno L. & Pendry J. B. Surfaces with holes in them: new plasmonic metamaterials. J. Opt. A: Pure Appl. Opt. 7, S97 (2005).

[b13] Shen X., Cui T. J., Martin-Cano D. & Garcia-Vidal F. J. Conformal surface plasmons propagating on ultrathin and flexible films. PNAS 110, 40–45 (2013). 23248311 10.1073/pnas.1210417110 PMC3538259

[b14] Quesada R., Martín-Cano D., García-Vidal F. J. & Bravo-Abad J. Deep-subwavelength negative-index waveguiding enabled by coupled conformal surface plasmons. Opt. Lett 39, 2990–2993 (2014). 24978255 10.1364/OL.39.002990

[b15] Gan Q., Fu Z., Ding Y. J. & Bartoli F. J. Ultrawide-Bandwidth Slow-Light System Based on THz Plasmonic Graded Metallic Grating Structures. Phys. Rev. Lett. 100, 256803 (2008). 18643690 10.1103/PhysRevLett.100.256803

[b16] Maier S. A., Andrews S. R., Martín-Moreno L. & García-Vidal F. J. Terahertz Surface Plasmon-Polariton Propagation and Focusing on Periodically Corrugated Metal Wires. Phys. Rev. Lett. 97, 176805 (2006). 17155495 10.1103/PhysRevLett.97.176805

[b17] Yu N. . Designer spoof surface plasmon structures collimate terahertz laser beams. Nat Mater 9, 730–735 (2010). 20693995 10.1038/nmat2822

[b18] Williams C. R. . Highly confined guiding of terahertz surface plasmon polaritons on structured metal surfaces. Nat Photon 2, 175–179 (2008).

[b19] Pors A., Moreno E., Martin-Moreno L., Pendry J. & Garcia-Vidal F. Localized Spoof Plasmons Arise while Texturing Closed Surfaces. Phys. Rev. Lett. 108, 223905 (2012). 23003598 10.1103/PhysRevLett.108.223905

[b20] Shen X. & Cui T. J. Ultrathin plasmonic metamaterial for spoof localized surface plasmons. Laser & Photonics Reviews 8, 137–145 (2014).

[b21] Liao Z., Pan B. C., Shen X. & Cui T. J. Multiple Fano resonances in spoof localized surface plasmons. Opt. Express 22, 15710–15717 (2014). 24977830 10.1364/OE.22.015710

[b22] Gao F. . Vertical transport of subwavelength localized surface electromagnetic modes. Laser & Photonics Reviews 9, 5 (2015).

[b23] Huidobro P. A. . Magnetic Localized Surface Plasmons. Phys. Rev. X 4, 021003 (2014).

[b24] Liao Z. . High-order localized spoof surface plasmon resonances and experimental verifications. Sci. Rep. 5, 9590 (2015). 25873523 10.1038/srep09590 PMC4397533

[b25] Gao Z. . Experimental demonstration of high-order magnetic localized spoof surface plasmons. Applied Physics Letters 107, 041118 (2015).

[b26] Haynes C. L. . Nanoparticle Optics: The Importance of Radiative Dipole Coupling in Two-Dimensional Nanoparticle Arrays^†^. J. Phys. Chem. B 107, 7337–7342 (2003).

[b27] Zhao L., Kelly K. L. & Schatz G. C. The Extinction Spectra of Silver Nanoparticle Arrays: Influence of Array Structure on Plasmon Resonance Wavelength and Width^†^. J. Phys. Chem. B 107, 7343–7350 (2003).

[b28] Zhang S., Genov D. A., Wang Y., Liu M. & Zhang X. Plasmon-Induced Transparency in Metamaterials. Phys. Rev. Lett. 101, 047401 (2008). 18764363 10.1103/PhysRevLett.101.047401

[b29] Gu J. . Active control of electromagnetically induced transparency analogue in terahertz metamaterials. Nat Commun 3, 1151 (2012). 23093188 10.1038/ncomms2153

